# Randomized and Blind Evaluation of the Efficacy of a Full-Spectrum Oral *Cannabis sativa* Oil Extract, Standardized Based on CBD-A, CBD and THC-A, THC in Canines with Chronic Osteoarthritis

**DOI:** 10.3390/ani16060900

**Published:** 2026-03-13

**Authors:** Escobar Torres Benjamin, Silva Elgueta Maria Teresa, Navarro Soto Alexander, Suárez Araya Stephanie, Sandoval Contreras Martín, Arrau Barra Sylvia

**Affiliations:** 1 Escuela de Medicina Veterinaria, Facultad de Medicina y Ciencias de la Salud, Universidad Mayor, La Pirámide 5750, Huechuraba 8580745, Chile; benjamin.escobart@mayor.cl (E.T.B.); drnavarrovet@gmail.com (N.S.A.); 2 Facultad de Medicina, Universidad San Sebastián, Lago Panguipulli 1390, Puerto Montt 5480000, Chile; mariateresa.silva@uss.cl; 3 Clínica Veterinaria Los Pinos, Calle Heraldo Orrego 3011, Local 2, Sector Los Pinos, Quilpué 2340000, Chile; estephanita@hotmail.com; 4 Centro Veterinario Pablo de Rokha, Pedro Prado 12697, La Pintana 8820000, Chile; sandoval.med.vet@gmail.com

**Keywords:** osteoarthritis, cannabidiolic acid, chronic pain, tetrahydronannabinolic acid, Cannabidiol, analgesia, full spectrum extract

## Abstract

Chronic osteoarthritis (COA) is a progressive and degenerative condition that causes joint inflammation and pain, often requiring long-term pharmacological management. Conventional treatments may lead to adverse effects, tolerance, and limited analgesic efficacy. This randomized, double-blind clinical trial evaluated the analgesic potential of a full-spectrum *Cannabis sativa* oil extract administered orally twice daily over six weeks in dogs with COA. Subjects were assigned to three groups: Cannabis, Placebo, and Control. Pain was assessed using the Canine Brief Pain Inventory (CBPI) and the Canine Osteoarthritis Staging Tool (COAST), which ranges from 0 to 4. The Cannabis extract (46.4 mg/mL) total cannabinoids: Cannabidiol (CBD), Cannabidiolic acid (CBDA), Delta-9-Tetrahydrocannabinol (Δ^9^-THC), and Tetrahydrocannabinolic acid (THCA), were administered using a cautious dose escalation protocol. Treatment began at ~0.1 mg/kg every 12 h, increasing by one drop (1.16 mg) every 72 h. This gradual titration continued until reaching the maximum tolerated dose (2 mg/kg every 12 h), which was maintained for the final two weeks. The protocol was designed to minimize adverse effects and allow close monitoring, especially in geriatric or clinically fragile dogs. By day 28, when the DMT was reached, the Cannabis group showed a 39.6% reduction in CBPI scores, compared to 24.7% in the Placebo group and a 1.6% increase in the Control group. COAST scores improved from level 4 to level 3 in 55.5% of dogs in the Cannabis group, with no changes observed in the other groups. We hypothesize that the co-administration of carprofen, meloxicam, or pregabalin with a full-spectrum *Cannabis sativa* extract—rich in acidic cannabinoids and terpenes—enhances pain relief and mobility in dogs with COA more effectively than conventional therapies alone. This study aimed to assess the efficacy of an oily full-spectrum *Cannabis sativa* extract as an adjunctive treatment to NSAIDs in twenty-seven dogs diagnosed with COA, and to compare pain intensity across three treatments groups.

## Simple Summary

This clinical study evaluated the safety and efficacy of a full-spectrum *Cannabis sativa* oil extract for managing chronic osteoarthritis (COA) pain in dogs. Over six weeks, dogs were randomly assigned to Cannabis, Placebo, or Control groups. The Cannabis extract, containing Cannabidiol (CBD), Cannabidiolic acid (CBDA), Delta-9-Tetrahydrocannabinol (Δ^9^-THC), and Tetrahydrocannabinolic acid (THCA), was administered orally in a dose escalation protocol up to 2 mg/kg. Pain and clinical progression were assessed using the Canine Brief Pain Inventory (CBPI) and the Canine Osteosteoarthritis Staging Tool (COAST). Results showed an important reduction in CBPI scores in the Cannabis group, compared to the Placebo group, and a slight increase in the Control group. Additionally, more than 50% of dogs in the Cannabis group improved one stage on the COAST scale, with no changes in the other groups, with no adverse effects observed. These findings support the use of oral full-spectrum Cannabis oil as a safe and effective complementary therapy for improving pain and quality of life in dogs with COA.

## 1. Implications

This study shows that *Cannabis sativa* oil extract can reduce pain in dogs with chronic osteoarthritis. Conventional treatments may cause adverse effects or lose effectiveness over time, but this research suggests that Cannabis therapy is a safe and effective adjunct.

Specifically, CBDA and THCA, along with their decarboxylated metabolites, were incorporated into a multimodal treatment approach, complementing traditional therapies. By improving mobility and comfort, this combined treatment enhances dogs’ quality of life. These findings may encourage further research into Cannabis-based pain relief for other animals and even humans, offering a promising alternative for managing chronic pain conditions.

## 2. Introduction

Canine Chronic Osteoarthritis (COA) is a progressive joint disease that significantly impairs quality of life due to chronic pain and reduced mobility [1]. Although it affects dogs of various ages, risk factors such as breed, obesity, trauma, and joint malformations increase susceptibility.

Non-steroidal anti-inflammatory drugs (NSAIDs) are widely used in COA management, yet no single agent has demonstrated consistent superiority in efficacy or safety [1,2]. Multimodal analgesic strategies, including combinations like carprofen with pregabalin, offer enhanced pain control [2], but evidence remains limited regarding their integration with cannabinoid-based therapies. Moreover, existing studies are constrained by small sample sizes and a lack of robust clinical trials evaluating full-spectrum Cannabis extracts in veterinary patients.

Cannabinoids, such as Cannabidiol (CBD) and Delta-9-Tetrahydrocannabinol (Δ^9^-THC), interact with CB1, CB2, and GPR55 receptors expressed in joint tissues affected by COA [3,4]. Elevated levels of endocannabinoids like anandamide (AEA) and 2-AG in inflamed joints suggest a modulatory role in pain and inflammation [4]. It was demonstrated that CBD improves comfort and activity in dogs with OA, while later work showed that acidic cannabinoids (Cannabidiolic acid (CBDA), Tetrahydrocannabinolic acid (THCA)) exhibit superior absorption and distinct pharmacological effects compared to their neutral forms [5,6].

Full-spectrum Cannabis extracts, which include cannabinoids, acidic precursors, terpenes, and flavonoids, may enhance therapeutic outcomes through synergistic interactions known as the entourage effect [7,8]. Terpenes such as β-myrcene, α-pinene, and β-caryophyllene contribute analgesic, anti-inflammatory, and neuroprotective properties [1,6,7]. The role of cannabinoid receptors in pain modulation was emphasized, supporting their relevance in chronic inflammatory conditions [8]. Additionally, [9] highlighted the importance of multimodal pain management in canine osteoarthritis, which aligns with the integrative approach adopted in this study, and demonstrated that Cannabidiolic acid from Cannabis acts as a selective cyclooxygenase-2 inhibitor, providing a biochemical basis for its potential role in pain modulation.

Pain assessment in veterinary medicine remains challenging due to its subjective nature. Validated tools such as the Canine Brief Pain Inventory (CBPI) and the COAST provide complementary frameworks for evaluating pain intensity and disease progression [2,10,11].

There is a lack of well-powered clinical trials evaluating the combined use of conventional analgesics and full-spectrum Cannabis extracts in dogs with COA. This study aimed to assess the efficacy of an oily full-spectrum *Cannabis sativa* extract as an adjunctive treatment to NSAIDs in twenty-seven dogs diagnosed with COA, and to compare pain intensity across three treatments groups

## 3. Material and Methods

### 3.1. Study Design

From a population of 70 dogs recruited by the Karü-Lawen Foundation (composed of veterinarians specialized in medical Cannabis), 27 dogs were selected to meet the following study requirements: male and female dogs, ≥7 years old; ≥8 kg, showing symptoms of COA, with progression of more than 6 months, clinically diagnosed with specialized radiological support, and either under or not under traditional pharmacological treatment.

Given the exploratory nature of this study, a non-probabilistic convenience sampling method was employed, selecting dogs with chronic osteoarthritis recruited by the Karü-Lawen Foundation. This strategy facilitated the inclusion of clinically homogeneous subjects, optimizing operational feasibility and access to confirmed cases. Importantly, the study design incorporated a Control group to enable comparative analysis, and although the overall sample size (*n* = 27) may limit statistical power, the approach is consistent with standards for preliminary veterinary research conducted under real-world constraints. The findings should be interpreted as hypothesis-generating, providing a basis for future controlled studies with larger and more representative populations

#### 3.1.1. Randomization and Group Allocation

A six-week randomized controlled trial was conducted to evaluate the effects of three distinct treatment strategies in dogs diagnosed with canine osteoarthritis (COA). Baseline data were collected from each participant, including the owner’s name, the dog’s name, age, sex, body weight, affected joint, and municipality. After screening for eligibility, selected participants were notified and enrolled in the study. Dogs were randomly assigned to treatment groups using a computer-generated randomization sequence created in Microsoft Excel, ensuring unbiased allocation and reproducibility. Random numbers were generated with a uniform distribution to provide equal probability of assignment across groups. All enrolled dogs were continuously monitored throughout the study by both the attending veterinarian and the owner.

The CBPI is a validated tool that assesses pain intensity and its impact on daily function in dogs with chronic osteoarthritis, using 10 owner-rated items on a 0–10 scale [2,10]. Complementing this, COAST classifies disease progression from 0 (no signs) to 4 (severe impairment), based on behavioral observations and clinical/radiographic findings [11]. Together, CBPI and COAST offer a comprehensive framework for evaluating pain and disease stage, guiding treatment decisions, and monitoring therapeutic outcomes [10,11]. The COAST scale was applied to stage the patients prior to treatment and during the progression of the pathology. The CBPI pain scale was also completed before treatment began, and weekly by the caregiver, in collaboration with the veterinarian. Before starting treatment, each dog underwent clinical, laboratory, and radiological evaluations by veterinary radiologists to confirm eligibility for inclusion in the study and to assess the clinical, laboratory, and radiological severity of their lesions. Two X-rays were taken in the standard projections of the affected limb, with a maximum effective period of 30 days.

The patients remained in their homes and continued with their regular lives throughout the study. Each dog was monitored by its attending physician with the assistance of the attending veterinarian, in addition to the attention of the tutor, who was responsible for recording noticeable changes throughout the week.

The Placebo oil used was also the same oil base (extra virgin olive oil, odorless, tasteless) added to the conventional treatment. In the case of the Cannabis oil plus conventional treatment and Placebo oil plus conventional treatment group, the caregiver did not know if the patient received Cannabis extract or not. All dogs underwent a one-week NSAID wash-out period before random assignment to treatment groups, based on clinical evaluations by veterinary specialists. All dogs enrolled in the study had previously received NSAID treatment; however, none achieved satisfactory pain control or improvement in quality of life, as determined by clinical evaluation, which qualified them for inclusion in the study.

#### 3.1.2. Therapeutic Protocols

(a) Previcox^®^: 4 mg/kg, SID, P.O., week 1. Week 2: 2.5 mg/kg PO SID, and from week 3 to week 6: 1.25 mg/kg PO SID and Pregabalin: 5 mg/kg, SID, PO.
(b) Meloxicam: 0.1 mg/kg, SID, P.O. and Pregabalin: 5 mg/kg, SID, PO.
(c) Carprofen: 4 mg/kg SID, P.O., from week 2 to week 6; 2.2 mg/kg, SID, P.O. and Pregabalin: 5 mg/kg, SID, PO., 6 weeks.

### 3.2. Randomization and Group Formation

The 27 selected patients were randomly distributed into three study groups of nine dogs each, to assess three treatments:

- Cannabis oil plus conventional treatment (treated group)
- Placebo oil plus conventional treatment.
- Conventional treatment

#### Validation and Quality Assurance

The validation and quality assurance of this study are based on a series of rigorous methodological criteria that ensure the reliability and accuracy of the results obtained. From experimental design to data analysis, processes have been implemented to minimize bias, optimize treatment safety, and reinforce the scientific solidity of the findings. Below are the key points that support the quality and validity of the study.

Randomized and double-blind design: This study was randomized and blinding minimized bias, ensuring that results are attributable to the treatment and no other factors.

Use of validated evaluation tools: The *CBPI* and *COAST* are established and reliable scales for measuring pain and disease progression.

Dose escalation protocol: Gradual dosing helps determine the optimal dose without compromising safety.

Monitoring of adverse reactions: The absence of reported side effects supports the treatment’s safety validation.

### 3.3. Analysis of the Oily Extract of C. sativa

#### Equipment (HPLC)

Waters HPLC equipment:

Degasser Serial No. M10DG2644M (Waters Corporation, Milford, MA, USA)
Binary Pump 1525 Serial Number K1025P263A (Waters Corporation, Milford, MA, USA)
UV-Visible Detector 2489 Serial No. M1087E218A (Waters Corporation, Milford, MA, USA)
Column Furnace Series No. C115CH760G (Waters Corporation, Milford, MA, USA).

The analysis was conducted using a Waters HPLC system equipped with a degasser (Serial No. M10DG2644M), binary pump 1525 (Serial No. K1025P263A), UV-Visible detector 2489 (Serial No. M1087E218A), and column furnace (Serial No. C115CH760G). The oily *Cannabis sativa* extract was diluted in ethanol, filtered through a 0.22 µm membrane, and injected into the system. Separation was achieved using a reversed-phase C18 column under isocratic conditions with a mobile phase consisting of acetonitrile and water (70:30 *v*/*v*). Detection was performed at 220 nm.

Calibration curves were constructed using certified analytical standards for THC, THCA, CBD, CBDA, and CBN, enabling accurate quantification across a broad cannabinoid profile. The method was validated for specificity, linearity, and reproducibility, and was deemed suitable for the reliable quantification of cannabinoids in complex biological matrices.

### 3.4. Cannabinoid Profile of Standardized Cannabis sativa Extract

The *Cannabis sativa* extract was standardized in an olive oil base and subsequently analyzed by Terpene Analytics, a laboratory that provided a detailed cannabinoid profile report. The resin was obtained using the Rick Simpson Oil (RSO) method, which involves diluting the dried plant material in ethanol, followed by controlled evaporation. This process yields full-spectrum resin rich in Cannabidiolic acid (CBD-A), with cannabinoids undergoing decarboxylation [12].

**Figure 1:**
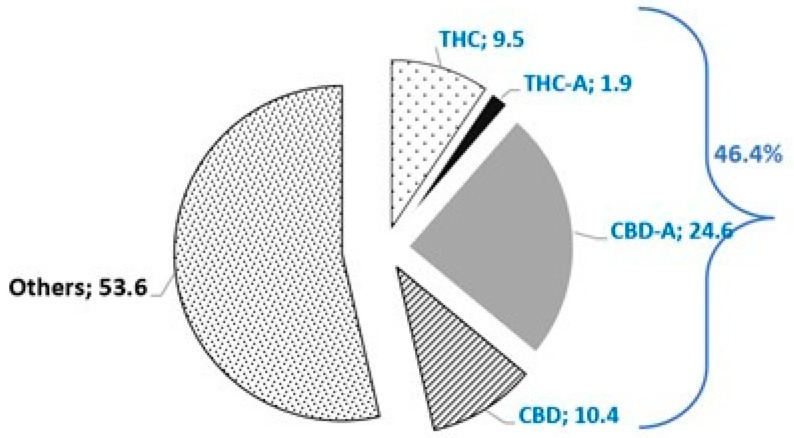
Percentage distribution of cannabinoids in the *Cannabis sativa* extract analyzed by Terpene Analytics. The extract contains detectable levels of Δ9-Tetrahydrocannabinol (THC), Δ9-Tetrahydrocannabinolic acid (THCA), Cannabidiol (CBD), and Cannabidiolic acid (CBDA).

Figure 1 illustrates the percentage distribution of cannabinoids present in the analyzed extract. Both acidic and neutral forms were identified, with CBD-A and CBD being the predominant compounds. Cannabinol (CBN) was not detected in the sample.

### 3.5. Drops Concentration and Treatment

Based on the analytical report, the concentrations of phytocannabinoids per drop (approximately 0.05 mL) were as follows: THC = 0.4725 mg, THCA = 0.09635 mg, CBDA = 1.2345 mg, and CBD = 0.51888 mg, resulting in a total of 2.32 mg of cannabinoids per 2-drop dose (0.1 mL). This corresponds to approximately 1.16 mg of total cannabinoids per drop, consistent with the formulation of a 10% oral resin preparation (1 g of resin in 10 mL of olive oil).

#### Description of the Dose Escalation Protocol in the Study

Due to the inability to accurately administer fractional drops, dose escalation was performed using whole drops only. Each drop contained approximately 2.32 mg of phytocannabinoids, based on a solution concentration of 46.4 mg/mL and an estimated standard of 20 drops per milliliter. The dose was increased every 72 h by one additional drop per administration, given twice daily, resulting in a 1.16 mg increase per dose. The escalation protocol consisted of nine incremental steps over a four-week period, starting at approximately 0.1 mg/kg and progressing to a maximum tolerated dose (DMT) of 2 mg/kg. This final dose was maintained during the last two weeks of the study. Table 1 summarizes the dose escalation process applied to nine canine patients, whose body weight ranged from 9 to 60 kg.

Although Table 1 shows that the two patients weighing 9 and 9.5 kg appear to exceed the DMT of 2 mg/kg, it is important to clarify that this value refers to the total daily dose (mg/kg/day). The dosing protocol involved twice-daily administration, meaning that each individual dose (every 12 h) remained within the 2 mg/kg limit.

Given the presence of Δ-9 THC and THCA, a slow and cautious titration schedule was implemented to minimize the risk of psychoactive side effects, particularly in geriatric or clinically fragile patients. In reference [13], escalating doses ranging from 18.3 to 640.5 mg of CBD per administration (~2–62 mg/kg) were used, with the second dose increasing by 2–2.5× and subsequent doses by 1.2–2×. Rather than directly extrapolating from previous studies, our protocol was adapted to the clinical context and therapeutic objectives of this trial. The rationale behind this dosing pattern reflects a commitment to safety, gradual pharmacological exposure, and individualized adjustment in veterinary patients. By limiting dose escalation, we aimed to optimize tolerability while still exploring the therapeutic potential of full-spectrum *Cannabis sativa* extracts in combination with conventional analgesics. This cautious titration strategy was selected to minimize the risk of adverse effects and to allow close monitoring of individual responses, particularly in geriatric dogs with chronic osteoarthritis.

### 3.6. Inclusion, Exclusion Criteria, and Characterization of Canine Subjects in the Chronic Osteoarthritis Clinical Study

All dogs included in the study were clinically diagnosed with COA, with the support of specialized radiological imaging, regardless of whether they were receiving conventional pharmacological treatment. Prior to enrollment, each subject underwent comprehensive clinical, hematological, and radiographic evaluations conducted by board-certified veterinary radiologists to confirm eligibility and to assess the severity of the condition. Two standard-projection radiographs of the affected limb were obtained within 30 days prior to treatment initiation.

Exclusion criteria included the presence of clinical or laboratory abnormalities attributable to conditions unrelated to COA, failure of owners to comply with informed consent requirements, undisclosed pregnancies, or non-adherence to the study protocol by the attending veterinarian. Throughout the study period, all patients remained in their home environments and continued their usual daily routines.

All enrolled dogs were over 7 years of age and included both males and females. Subjects were evenly distributed among the three study groups: Cannabis, Placebo, and Control. Baseline demographic and anthropometric characteristics are summarized in Table 2. Regarding body weight, the Cannabis group had a mean of 29.0 kg (SD = ±17.0), median of 27.0 kg, and a range of 9.0–60.0 kg, with an interquartile range (IQR) of 20.2. The Placebo group showed a mean of 23.9 kg (SD = ±6.5), median of 22.9 kg, range of 16.0–31.8 kg, and IQR of 12.0. The Control group had a mean weight of 25.3 kg (SD = ±10.1), median of 25.1 kg, range of 7.1–40.0 kg, and IQR of 5.6.

In terms of age, the Cannabis group had a mean of 9.2 years (SD = ±2.1), median of 9.0 years, range of 6.0–13.0 years, and IQR of 3.0. The Placebo group had a mean age of 10.4 years (SD = ±3.6), median of 11.0 years, range of 5.0–16.0 years, and IQR of 5.3. The Control group presented a mean age of 10.9 years (SD = ±2.5), median of 10.0 years, range of 7.0–14.0 years, and IQR of 3.0 (Table 2).

### 3.7. Statistical Analysis of Results

For data analysis, descriptive statistics were applied, including the number of cases, mean, median, and interquartile range (IQR), complemented by line graph visualizations to illustrate trends over time. To evaluate statistically significant differences in pain levels across treatment groups and assessment periods, a one-way analysis of variance (ANOVA) was performed. Post hoc comparisons were conducted using Scheffé’s test, following verification of normality with the Shapiro–Wilk test and homogeneity of variances. Comparisons of pain levels before and after reaching the DMT were conducted using paired-sample t-tests. Additionally, intergroup comparisons of pain levels were performed using standardized scores (z-scores), accounting for baseline differences in pain intensity. Statistical significance was set at *p* < 0.05.

## 4. Results

At baseline CBPI assessment, the Cannabis group showed a mean of 47 points (SD = ±8 units), the Placebo group 59 (SD = ±9 units), and the Control group 48 (SD = ±17 units). The medians and ranges were Cannabis 49 (30–57), Placebo 56 (47–73), and Control 42 (33–74), with interquartile ranges (IQR) of 7, 11, and 30, respectively. On day 28, the mean scores were Cannabis 31 (SD = ±14), Placebo 45 (SD = ±13), and Control 48 (SD = ±15); the medians and ranges were Cannabis 27 (11–52), Placebo 40 (29–68), and Control 40 (30–74), with IQRs of 18, 13, and 24, respectively. For the DMT, corresponding to the time point when the maximum tolerated dose was reached (week 1 and week 2), the values were Cannabis with a mean of 27 (SD = ±16), median of 24 (range: 1–55), and IQR of 15; Placebo with a mean of 40, median of 40 (range: 12–63), and IQR of 7; and Control with a mean of 47, median of 40 (range: 32–71), and IQR of 19.

These results are summarized in Table 3, which presents the progression of CBPI from baseline to day 28, as well as the values recorded at the point of maximum tolerated dose. In the initial COAST scale assessment, all groups showed identical values: mean of 4, standard deviation of 0, median of 4, range of 4–4, and IQR of 0.

**Figure 2:**
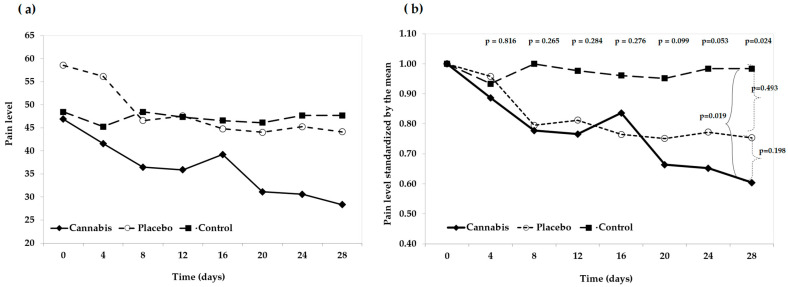
Pain level progression over time by treatment group. (**a**) Raw pain scores for the Cannabis, Placebo, and Control groups from day 0 to day 28. (**b**) Pain scores standardized by group mean, showing baseline equivalence and comparative trajectories.

To enhance the visualization of pain progression from baseline (day 0) to day 28, line graphs were constructed. As shown in in Figure 2a, pain scores decreased in both the Control and Placebo groups, while the Cannabis group exhibited relative stability over time. However, baseline pain levels differed across groups, potentially confounding the interpretation of treatment effects. To address this, data were standardized based on group means, allowing for a more accurate comparison of trajectories. The resulting visualization, shown in Figure 2b, demonstrates that all groups started under statistically comparable conditions, with no significant differences at baseline (F = 2.48; *p* = 0.10). No statistically significant differences were observed on days 4, 12, 16, 20, or 24. However, a significant difference was detected on day 28 (*p* = 0.024). Post hoc multiple comparisons revealed no significant differences between the Placebo and Control groups (*p* = 0.198), nor between the Placebo and Cannabis groups (*p* = 0.493). A statistically significant difference was found between the Cannabis and Control groups (*p* = 0.019), indicating a distinct treatment effect at the final time point.

**Figure 3:**
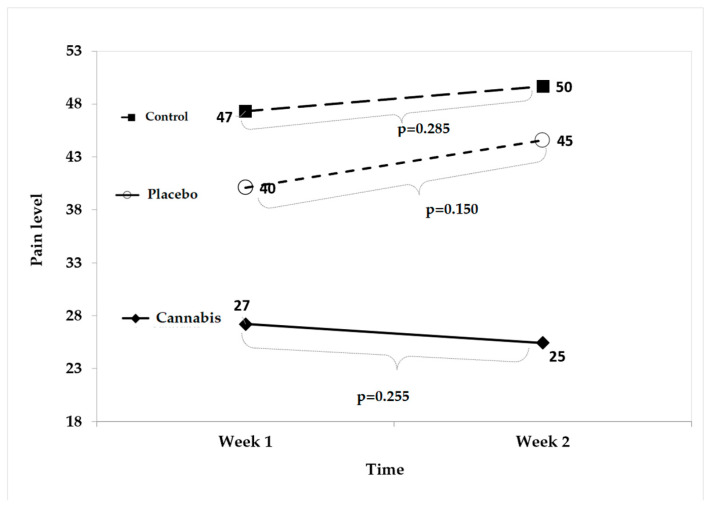
Comparison of pain levels between the Cannabis, Placebo, and Control groups one week after reaching the maximum tolerated dose. No statistically significant differences were observed (*p* > 0.05).

One week after reaching the maximum tolerated dose, pain levels were assessed across the Cannabis, Placebo, and Control groups. No statistically significant differences were observed between the groups (Cannabis vs. Placebo: *p* = 0.255; Cannabis vs. Control: *p* = 0.150; Placebo vs. Control: *p* = 0.285). These *p*-values indicate that no differential treatment effect could be attributed at this stage of the study. The corresponding results are presented in Figure 3.

## 5. Discussion

Our findings are consistent with recent analyses of commercial CBD oils, which highlight the significant variability in cannabinoid concentrations across products and underscore the importance of standardized extraction and quantification methods [14]. The use of full-spectrum RSO extracts, such as the one described here, provides a broad cannabinoid profile, including both acidic and decarboxylated forms, which may enhance therapeutic potential through the entourage effect.

It is important to note that although individual cannabinoid concentrations are reported, these compounds differ in chemical structure and molecular weight. Therefore, their percentages cannot be directly summed without applying a molecular conversion factor that accounts for the decarboxylation process. For example, the total THC-related content includes both THC and THCA, but their combined pharmacological potential must be interpreted with caution unless adjusted for molecular equivalence.

In this study, CBN was present at concentrations so low that it was considered non-detectable, making it unlikely to contribute meaningfully to any pharmacological effects under these conditions. This highlights the importance of accurate quantification when evaluating the therapeutic relevance of minor cannabinoids.

Separately, the relatively high THC potential (11.141%) emphasizes the need for precise dosing and safety monitoring, particularly in veterinary applications, where species-specific sensitivity and individual variability must be carefully considered.

To our knowledge, this is the first study conducted in Chile that evaluates the effects of Cannabis on pain in canines. We used extracts rich in CBDA, CBD, THC, and THCA at a maximum dose of 2 mg/kg, administered orally during the last two weeks of treatment. Patients did not experience any adverse reactions.

A significant analgesic response was observed by day 28 of treatment, coinciding with the administration of the maximum tolerated dose (MTD) of 2 mg/kg, at which patients reported optimal comfort levels [15]. This outcome aligns with previous studies employing a gradual dose escalation protocol, where Cannabis oil safety was assessed every four days across ten incremental steps [13]. In our study, however, the escalation was performed every three days. This adjustment was made in consideration of the relatively short duration of the study and the moderate nature of the dose increments, which were designed to balance safety with the need for timely therapeutic effects. Such titration strategies are clinically relevant, particularly in geriatric patients or those with comorbidities, as they allow for progressive adaptation to cannabinoids, potentially enhancing pain tolerance and reducing interference with daily activities effects that were reflected in both assessment scales used in this study. From a mechanistic perspective, the observed analgesic effects may be mediated by the activation of cannabinoid receptors CB1 and CB2, as well as TRPV1 channels, which are known to modulate nociceptive signaling and inflammatory responses. Additionally, neuroprotective and anti-inflammatory pathways triggered by CBD could contribute to the reduction in pain perception, although further studies are needed to confirm these mechanisms in veterinary patients [16].

It is important to note that the use of CBD-only oil excludes several bioactive compounds, including terpenes, flavonoids, and other cannabinoids such as THC and its biosynthetic precursors. These compounds may contribute synergistically to therapeutic effects via the “entourage effect.” In related studies, formulations containing a broader cannabinoid spectrum were administered transdermal at doses of 4 mg/kg of total cannabinoids, revealing that acidic precursors such as CBDA and THCA exhibit superior absorption profiles compared to CBD or THC [17]. This suggests that the pharmacokinetics and clinical efficacy of cannabinoid treatments may vary significantly depending on the route of administration and the chemical composition of the extract.

Among the limitations of this study, the exclusive use of CBD restricts the generalizability of the findings to full-spectrum formulations. Furthermore, the relatively small sample size and the absence of a Control group limit the statistical power and the ability to draw causal inferences. Variability in conventional treatments administered alongside cannabinoid therapy may also have influenced the outcomes, underscoring the need for standardized protocols in future research.

Three reviewed studies demonstrated a significant effect of CBD oil in reducing pain and improving activity levels in dogs, as reported through subjective pain and activity scoring systems by both owners and veterinarians [1,2,18]. These findings support the potential of CBD as a complementary analgesic in canine osteoarthritis (COA). However, a previous study [19] introduced objective gait analysis and found no significant improvement in locomotion among dogs treated with CBD oil compared to the Placebo group, highlighting the limitations of relying solely on subjective measures and the need for more robust, quantitative endpoints in future research. Currently, the evidence supporting the efficacy of CBD oil in alleviating COA-related pain in dogs, particularly when used alongside conventional treatments such as NSAIDs and other analgesics, remains inconclusive. In contrast, our study employed a broader cannabinoid profile including CBD, CBDA, THC, and THCA and yielded markedly positive outcomes in terms of pain reduction and functional improvement [5]. This suggests that multi-cannabinoid formulations may offer enhanced therapeutic benefits, possibly through synergistic interactions among the compounds.

The results contribute to and complement previous studies evaluating the toxicology and pharmacology of major cannabinoids, particularly THC and CBD [13,20]. Importantly, our findings underscore the relevance of biosynthetic precursors THCA and CBDA, which accounted for 1.9% and 24.6% of the total cannabinoid content, respectively. These acidic forms have been investigated in rodent and human models [8,9,21,22,23], and their inclusion in our formulation appears to have played a significant role in the observed clinical effects. Given their non-psychotropic nature and favorable safety profiles, CBDA and THCA warrant further investigation in veterinary medicine, particularly regarding their bioavailability, pharmacodynamics, and potential anti-inflammatory properties [24,25,26].

Recent research indicates that CBDA and THCA exhibit superior absorption in dogs compared to CBD, and that CBDA may enhance serum CBD levels by improving its absorption and retention [27,28,29]. This pharmacokinetic advantage could explain the enhanced efficacy observed in our study, despite the relatively low doses used. Additionally, the Placebo group showed a 25.05% reduction in pain, suggesting a possible analgesic and anti-inflammatory role of the lipid matrix. This effect mirrors findings in human studies on immune mediated inflammatory conditions such as rheumatoid arthritis, where phenolic compounds exert antioxidant, anti-inflammatory, and immunomodulatory actions. However, the absence of a Control group receiving sunflower oil alone limits our ability to isolate its specific contribution [30].

## 6. Conclusions

The complete extract of *Cannabis* sp., based on CBD-A, CBD and THC-A, THC with CBD predominance and maximum tolerated dose (MTD) of 2 mg/kg is effective and safe as a complementary treatment for COA for 6 weeks. However, more long-term studies, using full-spectrum extract of *Cannabis* sp., with higher doses and larger populations, are needed to identify the effects of *Cannabis* sp. over prolonged periods, as well as its effect in other species and against other pathologies that affect the quality of life of animals. Moreover, the relatively small sample size and the heterogeneity of conventional treatments administered alongside cannabinoids represent important limitations that should be addressed in future controlled trials.

## Figures and Tables

**Table 1:** Stepwise dose escalation protocol of phytocannabinoids in canine patients based on body weight (values in parentheses indicate number of drops administered per day).

Dog Weight (kg)	Initial Dose (mg)	Initial Dose (mg/kg)	Day 0	Day 3	Day 6	Day 9	Day 12	Day 15	Day 18	Day 21	Day 24	Day 27	Total Daily Dose (mg)	Total Daily Dose (mg/kg)
9.5	0.10	0.10	1.0 (1)	2.1 (2)	3.3 (3)	4.4 (4)	5.6 (5)	6.8 (6)	7.9 (7)	9.1 (8)	10.2 (9)	11.4 (10)	22.78	2.40
39.0	0.39	0.10	3.9 (3)	5.1 (4)	6.2 (5)	7.4 (6)	8.5 (7)	9.7 (8)	10.9 (9)	12.0 (10)	13.2 (11)	14.3 (12)	28.68	0.74
47.0	0.47	0.10	4.7 (4)	5.9 (5)	7.0 (6)	8.2 (7)	9.3 (8)	10.5 (9)	11.7 (10)	12.8 (11)	14.0 (12)	15.1 (13)	30.28	0.64
22.0	0.22	0.10	2.2 (2)	3.4 (3)	4.5 (4)	5.7 (5)	6.8 (6)	8.0 (7)	9.2 (8)	10.3 (9)	11.5 (10)	12.6 (11)	25.28	1.15
60.0	0.60	0.10	6.0 (5)	7.2 (6)	8.3 (7)	9.5 (8)	10.6 (9)	11.8 (10)	13.0 (11)	14.1 (12)	15.3 (13)	16.4 (14)	32.88	0.55
29.0	0.29	0.10	2.9 (3)	4.1 (4)	5.2 (5)	6.4 (6)	7.5 (7)	8.7 (8)	9.9 (9)	11.0 (10)	12.2 (11)	13.3 (12)	26.68	0.92
18.8	0.19	0.10	1.9 (2)	3.0 (3)	4.2 (4)	5.4 (5)	6.5 (6)	7.7 (7)	8.8 (8)	10.0 (9)	11.2 (10)	12.3 (11)	24.64	1.31
9.0	0.09	0.10	0.9 (1)	2.1 (2)	3.2 (3)	4.4 (4)	5.5 (5)	6.7 (6)	7.9 (7)	9.0 (8)	10.2 (9)	11.3 (10)	22.68	2.52
27.0	0.27	0.10	2.7 (2)	3.9 (3)	5.0 (4)	6.2 (5)	7.3 (6)	8.5 (7)	9.7 (8)	10.8 (9)	12.0 (10)	13.1 (11)	26.28	0.97

**Table 2:** Demographic and anthropometric characteristics of the experimental groups: Cannabis, Placebo, and Control.

Group	Statistic	Cannabis	Placebo	Control
Weight	Mean (SD)	29.0 (±17.0)	23.9 (±6,5)	25.3 (±10.1)
	Median (Range)	27.0 (9.0–60.0)	22.9 (16.0–31.8)	25.1 (7.1–40.0)
	IQR	20.2	12.0	5.6
Age	Mean (SD)	9.2 (±2.1)	10.4 (±3.6)	10.9 (2.5)
	Median (Range)	9 (6–13)	11 (5–16)	10 (7–14)
	IQR	3.0	5.3	3.0

**Table 3:** Progression of CBPI and COAST scores over time in Cannabis, Placebo, and Control groups.

Group	CBPI (Time days)								DMT (CBPI)		COAST	
	Initial	4	8	12	16	20	24	28	Week-1	Week-2	Initial	Final
**Cannabis (*n* = 9)**												
Mean (SD)	47 (±8)	42 (±8)	36 (±11)	36 (±12)	39 (±11)	31 (±14)	31 (±14)	28 (±13)	27 (±16)	25 (±14)	4 (0)	3 (±1)
Median (Range)	49 (30-57)	43 (29-52)	36 (21-51)	34 (21-54)	41 (23-52)	27 (15-55)	27 (11-52)	24 (9-52)	24 (1-55)	24 (5-54)	4 (4-4)	3 (3-4)
IQR	7	11	21	22	16	19	18	14	15	6	0	1
**Placebo (*n* = 9)**												
Mean (SD)	59 (±9)	56 (±14)	47 (±18)	48 (±19)	45 (±16)	44 (±16)	45 (±13)	44 (±14)	40 (±14)	45 (±17)	4 (0)	4 (0)
Median (Range)	56 (47-73)	55 (41-79)	43 (26-80)	43 (31-80)	40 (30-73)	45 (22-70)	40 (29-68)	47 (20-65)	40 (12-63)	41 (22-72)	4 (4-4)	4 (4-4)
IQR	11	25	24	17	12	17	13	13	7	28	0	0
**Control (*n* = 9)**												
Mean (SD)	48 (±17)	45 (±14)	48 (±18)	47 (±14)	47 (±13)	46 (±13)	48 (±15)	48 (±14)	47 (±13)	50 (±12)	4 (0)	4 (0)
Median (Range)	42 (33-74)	40 (31-70)	38 (32-81)	43 (31-69)	40 (32-68)	39 (32-69)	40 (30-74)	40 (32-74)	40 (32-71)	50 (35-70)	4 (4-4)	4 (4-4)
IQR	30	24	26	22	19	19	24	20	19	20	0	0

## Data Availability

The original contributions presented in this study are included in the article. Further inquiries can be directed to the corresponding author. The data collected in this study is available to other researchers without restrictions. The statistical and experimental designs can be consulted whenever required. The database is under the custody of the investigators and is accessible whenever needed.
